# Using runaway replication to express polyhydroxyalkanoic acid (*pha)* genes from a novel marine bacterium in enteric bacteria: The influence of temperature and phasins on PHA accumulation

**DOI:** 10.1371/journal.pone.0275597

**Published:** 2022-12-07

**Authors:** Dana Kolibachuk, Benjamin J. Ryder, Edward R. Lyons, Ariel Woolsey, Margaret K. Lopes, Keya Thakkar, Mayelin Pacheco Nunez, Michaela Duquenoy, Nevan R. Valente, Ashley Nieves

**Affiliations:** Biology Department, Rhode Island College, Providence, Rhode Island, United States of America; Karl-Franzens-Universitat Graz, AUSTRIA

## Abstract

While plastics have revolutionized our world, plastic waste has serious environmental and economic impacts. Polyhydroxyalkanoic acid (PHA) is a bacterial carbon and energy reserve shown to be both biodegradable and biocompatible and could potentially replace conventional plastics. However, cost-effective mass production remains elusive. Bacteria often accumulate PHA as cytoplasmic granules. PHA synthase creates the PHA polymer from acetoacyl-CoA monomers, while phasins are small multifunctional proteins that are found in abundance on the granule surface. The PHA synthase gene from a novel marine isolate, *Vibrio* B-18 (or B-18), was placed in the presence or absence of an upstream phasin gene in a runaway replication plasmid using polymerase chain reaction (PCR) technology. Plasmid gene expression may be induced chemically or thermally. Overexpression of the PHA genes was demonstrated by SDS-PAGE analysis, and microscopy was used to detect PHA accumulation in three different enteric bacteria (*Escherichia coli*, *Klebsiella aerogenes*, and *Shigella flexneri*). While the B-18 genes were clearly overexpressed at 41°C, PHA accumulation occurred more readily at the lower (30°C) non-inducing temperature regardless of chemical induction if the phasin gene was present. A mutational analysis confirmed the identity of the start codon for the PHA synthase gene and provided evidence supporting the requirement for phasins to allow for PHA accumulation in the recombinant hosts. The findings described in this study confirm the conclusions obtained from related studies from other laboratories and lend support to the importance of including a phasin gene in addition to the basic genes needed for PHA synthesis and accumulation in recombinant enteric bacteria, such as *Escherichia coli*, *Klebsiella aerogenes*, and *Shigella flexneri*.

## Introduction

Plastics are synthetic organic polymers and the vast majority are derived from fossil hydrocarbons. Many of the commonly used plastics are not biodegradable. Large-scale plastic production began in 1950 and it has been estimated that 8300 million metric tons of virgin plastic were produced as of 2017. An estimated 6300 million metric tons of plastic waste was generated by 2015, with most of this accumulating in landfills and the natural environment [1]. Nearly 100 million metric tons can be found in the oceans with much of it traveling from land-based sources through estuaries [reviewed in 2]. If current trends continue, the amount of plastic waste is expected to nearly double by 2050 [1]. At this time, the production and use of plastics will use 20% of the total global oil supply, account for 15% of the total carbon budget [reviewed in 3], and the total amount of plastic waste in the oceans is expected to exceed the total amount of fish [4]. There has been extensive investigation into alternate materials, such as biopolymers from prokaryotes [recently reviewed in 5], that could substitute for conventional plastics yet are biodegradable.

Polyhydroxyalkanoic acids (PHAs) are polyesters produced by some prokaryotes when conditions become limiting for certain resources, such as nitrogen, while carbon sources remain plentiful. Many cells accumulate PHA as cytoplasmic inclusions or granules that are utilized as a carbon and energy source once the exogenous carbon sources become limiting. While PHA has been long known for its role as a carbonosome [6], recent findings have shown the presence of PHA granules in prokaryotic cells enhances survival against a variety of environmental stresses such as heat shock, low temperatures and freezing, oxidative pressure, osmotic shock, and exposure to heavy metals and ultraviolet light [reviewed in 7].

Since the discovery that some PHA has similar properties to polypropylene [8], PHA has been extensively researched as a potential replacement for petroleum-based plastics. Long known for its biodegradability in soil [reviewed in 9], research has shown that PHA may also biodegrade in a wide range of marine environments [10]. In addition, it is biocompatible, modifiable, and elastomeric making it suitable for many biomedical applications, including those on a nanoscale [reviewed in 5, 11, 12]. Recent studies have also investigated the potential for PHA as a biofuel [reviewed in 13]. Many strategies involving scientists from a variety of disciplines have been investigated to allow for cost-effective methods for PHA production [reviewed in 14, 15]. Despite extensive research over the past several decades, there are currently only thirteen manufacturers of PHA products [15]. The small number of manufacturers most likely reflects the production costs of PHA, which are estimated to be three to four times higher than that of conventional petroleum-based polymers [14].

Although many bacteria are known to accumulate PHA, PHA synthesis and accumulation is probably best understood in the gram-negative soil bacterium *Cupriavidus necator* [16], which has been reclassified several times [most recently, 17]. A minimum of three PHA-specific enzymes are required for the synthesis and accumulation of poly(3-hydroxybutyrate), a common form of bacterial PHA, in *Escherichia coli* [18]. Acetoacetyl-CoA is created from the condensation of two acetyl-CoA molecules by a PHA-specific 3-ketothiolase, encoded by the *phaA*_*Cn*_ gene. These are reduced by an NADPH-dependent acetoacetyl CoA reductase, encoded by *phaB*_*Cn*_, to form D-(-)-3-hydroxybutyryl-CoA, which is a PHA monomer. PHA synthase, encoded by *phaC*_*Cn*_, links two monomers together or links a monomer onto a growing PHA polymer through the formation of ester bonds. PHA of varying composition results from the substrate specificity of PHA synthase as well as from the available hydroxyacyl-CoA monomers present within the cell. Over 100 different monomers can be found in PHA [19, 20] and these monomers could be produced from other metabolic pathways, such as the fatty acid degradation pathway [for example, 21, 22].

In addition to PHA synthase and the available substrates, it was shown that alternate 3-ketothiolases can influence the PHA composition as the enzyme encoded by *phaA*_*Cn*_ was found to be specific for acetyl-CoA [reviewed in 16].

PHA synthases have been placed into four groups based on the amino acid sequence, substrate specificity, and the number of subunits [reviewed in 23, 24]. Type I synthases function as homodimers and contain many conserved regions with type III and IV synthases. Both of these latter synthases function as heterodimers. Type III heterodimers consist of PhaC and PhaE, while type IV heterodimers consist of PhaC and PhaR. Similar to type I, type II synthases are homodimers composed of either PhaC1 or PhaC2. Type I, III, and IV synthases prefer short-chain-length monomers, 3–5 carbons in length, while type II synthases prefer longer chain length monomers, ranging from 6–14 carbons, as substrates. Key amino acids required for the substrate specificity of type II PHA synthases were identified by mutational analyses [25]. In particular, specificity for 3-hydroxybutyrl-CoA was shown to be directly related to the amino acid residue at position 484. Type I and III synthases predominately contain valine or isoleucine, while type II synthases contain leucine at this position.

There are other PHA-specific proteins and many of these can be found on the surface of PHA granules [reviewed in 26]. In addition to PHA synthase, PHA depolymerases, phasins, and regulatory proteins comprise the four different types of granule-associated proteins. Of these, phasins are the most abundant and are believed to form network-like covers on the PHA-granule surface to control the number, size, and distribution of the granules within cells [for a recent review, see 27].

This study utilizes PHA genes from a bioluminescent marine bacterium isolated from a seawater sample collected from Buckroe Beach, Virginia. Prior to this study, this organism was shown to accumulate PHA containing monomers between four to six carbons in length (Kolibachuk & Dennis, unpublished results). Several attempts by outside vendors were made to identify this isolate. Initially, a fatty acid profile analysis (Microbial ID, Inc.) showed a good match to *Vibrio parahaemolyticus* GC subgroup B. A genomic analysis comparing the 16S RNA gene sequences (Accugenix, Inc.) showed this isolate to be only 0.93% different from *Vibrio alginolyticus*. However, it did not cluster directly with this organism. Therefore, we decided to call this isolate *Vibrio* B-18 (or B-18). A genomic DNA fragment from B-18 showing homology with a portion of the *phaC*_*Cn*_ gene was cloned into an *E*. *coli* plasmid. This study began with a DNA sequence analysis of the cloned fragment, which revealed the presence of open reading frames showing homology with *pha* genes such as those for PHA synthase and phasins. Noting that the close proximity of the phasin gene to *phaC* was not seen in a wide variety of bacteria [28, more recently 29], we hypothesized that the phasin gene was important for PHA synthesis and accumulation. To test this hypothesis, DNA fragments containing the *phaC*_*B18*_ gene in the presence and the absence of an upstream phasin gene (*phaP*_*B18*_) were subcloned into a runaway replication plasmid. With these constructs, plasmid replication is dependent upon the incubation temperature. At lower temperatures, the plasmids exist at low numbers in the cell and plasmid gene expression is minimal. Higher temperatures cause excessive plasmid replication. The increased plasmid load forces the cell to preferentially express plasmid genes [for example 30–36]. In addition, chemical induction may also be employed to overexpress the genes carried on the plasmid [for example, 34].

## Materials and methods

### Bacterial culture conditions

Table 1 lists the bacteria used in this study. For routine culture maintenance, the enteric bacteria (*E*. *coli*, *K*. *aerogenes*, and *S*. *flexneri)* were grown in Luria-Bertani medium (LB; Difco) supplemented with the appropriate antibiotics for plasmid maintenance. The final antibiotic concentrations are chloramphenicol (Cm) 25 μg/mL, tetracycline (Tc) 10 μg/mL, and ampicillin (Ap) 100 μg/mL. Unless indicated otherwise, cultures were incubated at 37°C. For agar plates, 15 g/L of agar was added.

**Table 1:** Bacterial strains and plasmids used in this study.

Strain or Plasmid	Important Features (reference or source)
*Vibrio* B-18	Source of *phaP*_*B18*_ and *phaC*_*B18*_ (isolated from Virginian coastline by D. Kolibachuk)
*E*. *coli* XL1-Blue	F’ Tn*10* (Tc^r^); *lac* for blue-white screening (Agilent)
*E*. *coli* DH5α	Strain for plasmid maintenance (Gibco-BRL)
*K*. *aerogenes* KC2671	General *Klebsiella* strain [37]
*S*. *flexneri* serovar 2b group B	General *Shigella* strain (ATCC 12022)
pPM9	Original clone containing 5.6-kb B-18 insert into pBBR1MCS-1 [38]; Cm^r^ (D. Dennis & P. Marzban)
pBluescript II SK (+)	Vector for Constructing Subclones; Ap^r^ (Agilent)
pJM9485	Runaway replication vector containing *lacZ*; Cm^r^ [34]
pDKF2	pJM9485 subclone containing *phaC*_*B18*_
pDKE6	pJM9485 subclone containing *phaP*_*B18*_*-phaC*_*B18*_
pDK108	*phaC*_*B18*_ nonsense mutation in pE6: first 2 codons were replaced with stop codons
pDK201	*phaC*_*B18*_ missense mutation in pE6: second Met codon replaced with an Ala codon
pDK401	*phaC*_*B18*_ silent mutation in pE6: second Met codon is retained while possible 2^nd^ Shine-Dalgarno sequence is removed
pDK602	*phaP*_*B18*_ nonsense mutation in pE6: replaced the first 3 codons with stop-stop-ala
pDK702	*phaP*_*B18*_ nonsense mutation in pE6: alters the first 5 codons with stop-pro-ala-asp-leu
pDK802	*phaP*_*B18*_ frameshift mutation in pE6: G insertion after the 14^th^ nucleotide results in a 9-amino acid peptide (M-Y-T-D-L-L-Q-N-F) with predicted size 1144 Da
pUMS	contains *C*. *necator phaA* and *phaB genes;* Tc^r^ [39]

For promoting PHA production, bacteria were grown on M9 medium [40] supplemented with 0.5% glucose, 0.1% nutrient broth powder, and the appropriate antibiotics needed for plasmid maintenance. If chemical induction was to be employed, IPTG (isopropyl-β-D-thiogalactopyranoside) was added to a final concentration of 1 mM. If thermal induction was to be employed, the cultures were incubated at 41°C. A temperature of 30°C was used for those cultures that were not subjected to thermal induction.

In every case, broth cultures were incubated with aeration at 125 rpm in an orbital shaker.

### DNA manipulation and electroporation

Standard techniques [40, 41] were used to manipulate DNA using commercially available enzymes (NE Biolabs) and kits for purification (Qiagen Corporation). Electrocompetent *E*. *coli*, *K*. *aerogenes*, and *S*. *flexneri* were prepared according to standard procedures originally used for *E*. *coli* [41]. Samples containing plasmid DNA were purified and concentrated in spin columns (QIAquick; Qiagen Corporation) prior to any electroporation experiment. The purified plasmids were placed into the electrocompetent cells using 0.1 cm gap cuvettes and a BioRad Gene Pulser set at 1.8 kV (“EC1” setting). The presence of the plasmids within the electroporated cells was confirmed by restriction endonuclease digestion of isolated plasmids followed by separation in 1% agarose-TAE. The resulting agarose gels were stained with ethidium bromide and visualized in an UV transilluminator.

### DNA sequence analysis of a cloned fragment from *Vibrio* B-18

Prior to this study, plasmid pPM9 was constructed and identified using methods that have been described previously [37]. Briefly, a B-18 genomic library was constructed by inserting 5 to 7-kb *Bam*HI fragments into the broad host range plasmid pBBR1MCS-1 [38]. *E*. *coli* XL1-Blue cells containing this library were screened using a digoxygenin-labeled PCR product corresponding to bases 1865 to 2430 of the *phaC* gene from *C*. *necator* [42]. In this study, a restriction map of the 5.6-kb insert was created and used to direct the creation of a series of overlapping subclones in pBluescript SKII+. These subclones were sequenced in both directions using an outside vendor (MWG Biotech Incorporated; High Point, NC) and a contiguous DNA sequence was constructed from overlapping sequence data using computer software (MacDNAsis). Sequence comparisons and alignments were performed used the Basic Local Alignment Search Tool (BLAST; National Center for Biotechnology Information).

The nucleotide sequence of a 2842-bp fragment containing B-18 genes involved in PHA synthesis can be found under GenBank accession number AY046537. The nucleotide sequence of the complete 5549-bp B-18 fragment is found under accession number MZ361737.

### Construction of runaway expression vectors containing B-18 genes

The DNA sequence of the cloned B-18 insert was used to devise several primers (Table 2) that amplify DNA sequences containing either *phaC*_*B18*_ alone (del3.4 and VpR3) or *phaP*_*B18*_*-phaC*_*B18*_ (del2.3 and VpR3). The PCR products were cloned using the Stratagene Ultra Blunt PCR Cloning Kit (Agilent Technologies) according to the manufacturer’s directions. Plasmids were isolated from white colonies on agar plates containing X-Gal (5-bromo-4-chloro-3-indolyl-β-D-galactopyranoside; final concentration 20 μg/mL) and screened with *Bam*HI to confirm the presence of insert DNA. The orientation and the identity of the insert DNA was further confirmed by DNA sequence analysis using an outside vendor (Rhode Island Genomics & Sequencing Center; University of Rhode Island). Plasmids containing the transcriptional start of the cloned genes oriented towards the *Bam*HI site/T7 primer of the cloning vector supplied with the kit (pSC-B-amp/kan) were selected for further subcloning into the runaway replication vector, pJM9485. These plasmids and pJM9485 were digested with *Bam*HI and *Sal*I and the resulting fragments were separated by electrophoresis in 1% agarose-TAE. DNA fragments corresponding to the pJM9485 vector (6 kb) and the inserts from the PCR clones (~ 1.9 kb for *phaC*_*B18*_ and ~2.3 kb for *phaP*_*B18*_*-phaC*_*B18*_) were gel purified and ligated with T4 DNA ligase. The ligation mixtures were placed into *E*. *coli* XL1-Blue by electroporation and plated onto LB-Cm agar containing X-Gal. Plasmid DNA was isolated from white colonies, and the presence of the inserts was confirmed by both restriction endonuclease digestion and DNA sequence analysis using an outside vendor (RI-GSC; University of Rhode Island). This procedure effectively replaced the 4-kb fragment containing *lacZ* and *lacY* in pJM9485 with the B-18 PCR products in the proper orientation with respect to the *tac* promoter in pJM9485. Using this procedure, plasmids pDKF2 and pDKE6 were constructed and respectively contain *phaC*_*B18*_ and *phaP*_*B18*_*-phaC*_*B18*_.

**Table 2:** Primers used to construct plasmids containing B-18 genes by PCR technology.

Primer	Sequence	Significance
del2.3	5’-GGTTGTAAAAGCAGTTAAGTAG-3’	located 65 bases upstream from *phaP*_*B18*_ translational start; forward primer
del3.4	5’-GGCACGGCTGTGGTTGTAAA-3’	located 37 bases upstream from *phaC*_*B18*_ translational start; forward primer
VpR3	5’-GGCGACCCTATAAGCCGCC-3’	located 44 bases downstream from *phaC*_*B18*_ translational stop codon; reverse primer
QCL1	5’-CCGAATATAGGAGTAAGACT**TAGTAA**CAGCACTTCTTTTCGG-3’	replaces *phaC*_*B18*_ codons 1 & 2 for two stop codons
QCL2	5’-CCGAAAAGAAGTGCTG**TTACTA**AGTCTTACTCCTATATTCGG-3’	replaces *phaC*_*B18*_ codons 1 & 2 for two stop codons
QCL3	5’-CCTTTGAATAAGGCC**GCG**CAAGAAGTGAACTTCG-3’	replaces 2^nd^ met codon in *phaC*_*B18*_ for an ala codon
QCL4	5’-CGAAGTTCACTTCTTG**CGC**GGCCTTATTCAAAGG-3’	replaces 2^nd^ met codon in *phaC*_*B18*_ for an ala codon
QCL7	5’-CGTGAATTCTCCT**CTAAATAAAGCC**ATGCAAGAAGTGAACTTCG-3’	replaces potential Shine-Dalgarno by 2^nd^ met codon in *phaC*_*B18*_ with degenerate codons
QCL8	5’-CGAAGTTCACTTCTTGCAT**GGCTTTATTTAG**AGGAGAATTCACG-3’	replaces potential Shine-Dalgarno by 2^nd^ met codon in *phaC*_*B18*_ with degenerate codons
QCL9	5’-CTCTAGGAGATAAACC**TAGTAAGCG**GATTTCTTC-3’	replaces first 3 codons of *phaP*_*B18*_ with stop-stop-ala; forward primer
QCL11	5’-GGAGATAAACC**TAGCCCGCCGACCTC**TTCAAAACTTTTAGCG-3’	replaces first 5 codons of *phaP*_*B18*_ with stop-pro-ala-asp-leu; forward primer
QCL13	5’-CCATGTACACTGATTT**G**CTTCAAAACTTTTAGCG-3’	G insertion after 14^th^ nucleotide in *phaP*_*B18*_; forward primer

Note: bold font indicates changes from the original sequence.

### Constructing mutations in either *phaP*_*B18*_ or *phaC*_*B18*_ in the runaway replication vector, pDKE6

Mutations in the *phaC*_*B18*_ gene of pDKE6 were constructed using the QuickChange Lightning Site-Directed Mutagenesis kit (Agilent Technologies) according to manufacturer’s directions. These kits make use of complimentary primers containing the mutation (highlighted in red in Table 2) in PCR experiments using pDKE6 as the template DNA to construct copies of the template plasmid containing the mutations found within the primers. In this kit, the PCR reaction mixtures were treated with *Dpn*I to remove the template DNA. The treated PCR products were placed into *E*. *coli* XL1-Blue by electroporation as described above. The electroporation mixtures were plated on LB-Cm plates containing X-Gal. Plasmid DNA was isolated from white colonies and the presence of the mutation was confirmed by DNA sequence analysis using an outside vendor (RI-GSC; University of Rhode Island). In this way, three mutations were created in the *phaC*_*B18*_ gene. Plasmid pDK108 was constructed with primers QCL1 and QCL2, pDK201 was constructed with primers QCL3 and QCL4, and pDK401 was constructed with primers QCL7 and QCL8.

Constructing mutations within *phaP*_*B18*_ using the QuickChange Lightning kit proved to be more challenging. Instead, the Stratagene Ultra Blunt PCR Cloning kit was employed in a similar procedure used to create both pDKE6 and pDKF2. In these experiments, QCL9, QCL11, or QCL13 were used as the forward primers with VpR3 as the reverse primer in PCR experiments to ultimately create pDK602, pDK702, or pDK802, respectively. The presence of the mutations was confirmed by a DNA sequence analysis performed by an outside vendor (RI-GSC; University of Rhode Island).

### Overexpression of the B-18 cloned gene products

In these studies, a temperature of 30°C was used as the non-inducing temperature. In all cases, fresh stock plates were prepared from freezer stocks and all cultures were grown at 30°C prior to the induction experiments. An overnight culture was used to inoculate 100 mL of LB-Cm in a 500-mL flask to an optical density at 600 nm wavelength (OD^600^) of 0.1. The inoculated flask was incubated at the non-inducing temperature with shaking at 125 rpm until the OD^600^ reached between 0.2–0.4. At this time, a 1-mL sample was taken, and the cells were harvested by centrifugation in a microcentrifuge. The cell pellet was resuspended in 0.333 mL of SDS-PAGE sample buffer, creating samples that were 3x concentrated. This was the time zero sample. At this time, 25-mL aliquots were placed into pre-warmed sterile 250-mL flasks. If chemical induction was to be considered, IPTG was added to a final concentration of 1 mM. These flasks were incubated with shaking at 125 rpm at either 30°C for uninduced samples or at 41°C for thermally induced samples. At regular timed intervals (1.5 h, 3.0 h post-induction), samples were taken and used to measure the OD^600^ and to prepare a 3x concentrated protein sample for SDS-PAGE analysis. At the end of the experiment, the SDS-PAGE samples were boiled for 5 minutes and stored frozen at -20°C. Samples were collected and analyzed by SDS-PAGE as described below on at least two separate occasions.

### Sodium dodecyl sulfate polyacrylamide gel analysis (SDS-PAGE)

The procedure used for SDS-PAGE analysis was based upon standard techniques [40]. The stored SDS-PAGE samples from above were boiled for 5 minutes prior to loading on a 1.5 mm thick discontinuous SDS-PAGE gel consisting of 4.5% acrylamide in the stacking gel and 12.5% acrylamide in the resolving gel. The volume of the sample was adjusted based on the optical density measurement so that an equivalent amount of protein was analyzed in each lane. An unstained SDS-PAGE protein standard (low range, Bio-Rad, Hercules, CA) was added to the wells flanking the sample lanes. Proteins were separated by SDS-PAGE using the Amersham Sturdier electrophoresis cell with a Bio-Rad PowerPac 300 power supply. Proteins were visualized by staining with Coomassie brilliant blue R-250 and destained gels were reproduced using the Chemi-Doc MP imaging system (BioRad).

### Detection of PHA granules by microscopy

With the enteric bacteria (*E*. *coli*, *K*. *aerogenes*, and *S*. *flexneri)*, it was necessary to place the runaway replication plasmids containing the B-18 genes into cells containing pUMS in order to supply *phaA*_*Cn*_ and *phaB*_*Cn*_ to allow for PHA accumulation. In the case of *E*. *coli*, DH5α (pUMS) was employed as pUMS carries the Tc resistance gene and could not be maintained in strain XL1-Blue.

Initially an overnight culture was prepared in rich medium from fresh stock cultures as described above. Cells were harvested by centrifugation and were resuspended in the equivalent volume (50 mL) of minimal medium supplemented as appropriate with antibiotics and IPTG followed by incubation at 30°C or 41°C with aeration at 125 rpm. The cultures were fed daily with 1 mL of 25% glucose in order to ensure that the carbon source remained plentiful throughout the experiment.

Heat-fixed bacterial smears were prepared in duplicate on glass slides each day for up to a total of four days. These were stained for PHA using Sudan Black or simple stained with safranin for 30 seconds using standard methods [40, 41]. These were observed under oil immersion (1000x total magnification) using a Leica DM750 compound light microscope equipped with a camera. These images were captured and enhanced using the Leica Acquire software. Each experiment was performed on at least two separate occasions.

## Results

### DNA sequence analysis of the cloned fragment

**Fig 1:**
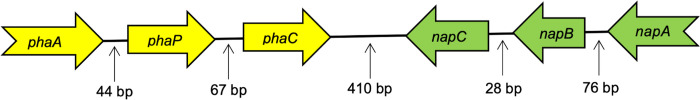
Map of the complete 5549-bp *Bam*HI fragment containing PHA synthesis genes from *Vibrio* B-18. The *pha* genes are involved in PHA synthesis: *phaA* encodes a PHA-specific 3-ketothiolase, *phaP* encodes a phasin, and *phaC* encodes PHA synthase. The *napA-C* genes encode proteins involved in the periplasmic reduction of nitrate. The large horizontal arrows indicate the orientation of the genes, while the small vertical arrows indicate the distance between the genes. The notched arrows indicate partial genes. This figure is not drawn to scale.

The complete 5549-bp sequence of the cloned *Bam*HI fragment from *Vibrio* species B-18 contains two divergently transcribed units (Fig 1), both of which lack a promoter and contain at least three genes. In each transcriptional unit, the spacing between the genes (28–76 bp) and the lack of sequence with homology to known promoters suggests that each transcriptional unit is an operon. As seen in the figure, the *phaC* gene is the last gene in an operon containing a partial gene with homology to *phaA* and an intact gene with homology to *Vibrio*-type phasins, called *phaP*, located between *phaA* and *phaC*. This DNA fragment contains no other PHA-specific genes.

**Fig 2:**
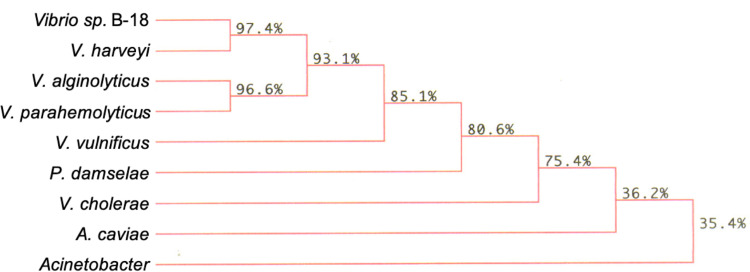
Phylogenetic tree of the *phaC* gene product from *Vibrio* B-18 from BLAST and Higgins Sharp alignment (MacDNAsis). The numbers indicate the percent identity in the aligned proteins. Genbank protein identification numbers: *Acinetobacter* species- AAA99474.1; *A*. *caviae-* BAA21815.1; *P*. *damselae-* ARR48929.1; *V*. *alginolyticus-* ALR94500.1; *V*. *cholerae-* APF84881.1; *V*. *harveyi-* AIV08552.1; *V*. *parahaemolyticus-* AHJ01595.1; and *V*. *vulnificus-* ANN29229.1.

As summarized in Fig 2, alignment of the *phaC*_*B18*_ gene product with other PHA synthases show it to be closely related to mostly uncharacterized PHA synthases from the genus *Vibrio* and more distantly related to the PHA synthases from *Aeromonas caviae* and *Acinetobacter*. Closer examination of the amino acid sequence reveals that PhaC_B18_ contains a valine at position 484 (results not shown). Given that only one PHA synthase gene is contained on this fragment and that the amino acid sequence shares many residues with type I synthases, one could conclude that this enzyme is an example of a type I synthase. A BLAST search of the protein encoded within *phaP*_*B18*_ revealed it to be a member of the phasin 2 superfamily, with 88–99% identity with putative phasins from members of the genus *Vibrio* (results not shown).

Because the PHA genes in the original cloned fragment lack promoters (Fig 1), it was necessary to subclone these genes to allow for expression studies. DNA fragments containing either *phaC*_*B18*_ or *phaP*_*B18*_*-phaC*_B18_ were cloned using PCR technology and ultimately placed into pJM9485 to create pDKF2 and pDKE6, respectively. The B-18 genes were placed in the correct orientation with respect to the *tac* promoter in pJM9485. In addition to chemical induction with IPTG, pJM9485 allows plasmid gene expression to occur by runaway replication, a method of gene expression where the plasmid copy number is controlled by the incubation temperature. Higher temperatures, such as 41°C, cause extensive plasmid replication. The high plasmid copy number forces the cell to preferentially express the genes carried on the plasmid [34].

### Gene expression and demonstration of runaway replication

**Fig 3:**
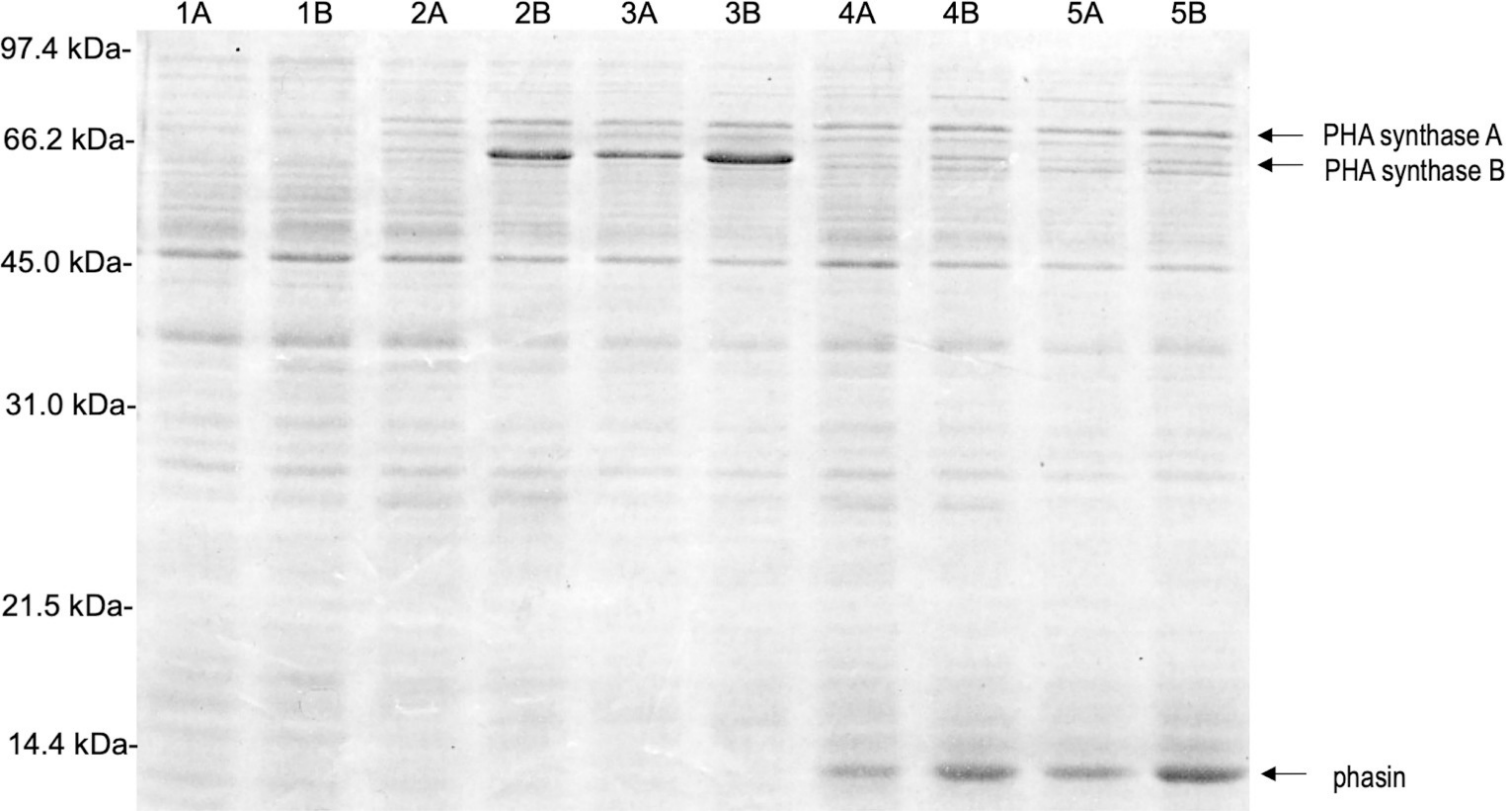
Comparison of chemical and thermal induction in *E*. *coli* XL1-Blue using SDS-PAGE analysis. Sample 1: (pDKE6) 30°C, 2: (pDKF2) 41°C, 3: (pDKF2) 41°C + IPTG, 4: (pDKE6) 41°C, and 5: (pDKE6) 41°C + IPTG. A = 1.5 hours post induction, B = 3.0 hours post induction. The sample volume was adjusted so that each lane contained the same amount of protein. The arrows indicate the probable location of PHA synthase (67.306 kDa or 62.675 kDa depending upon the location of the translational start) and the phasin (13.153 kDa predicted size).

The results from an SDS-PAGE analysis of the total protein from *E*. *coli* containing either pDKF2 (containing *phaC*_*B18*_) or pDKE6 (containing *phaP*_*B18*_*-phaC*_B18_) are shown in Fig 3. Samples were grown to an OD^600^ of approximately 0.2 prior to induction at 41°C in the presence or absence of the chemical inducer IPTG. The arrows in the figure show locations of induced proteins that correspond to the predicted sizes for both PHA synthase and the phasin. The “A” lanes in this figure contain samples taken at 1.5 h post-induction while the”B” lanes contain samples taken at 3.0 h post-induction. The first two lanes contain samples from cells carrying pDKE6 that continued incubation at 30°C and serve as a control for this experiment. A similar banding pattern was seen in uninduced cells carrying pDKF2 and for the time zero samples (results not shown in the figure). Lanes 2A-3B contain samples taken from cells containing pDKF2, while lanes 4A-5B contain samples from cells containing pDKE6.

In the lanes containing induced samples, the protein bands indicated by the arrows increase in intensity with the longer incubation time (for example, compare lane 2A to lane 2B) and the addition of IPTG seems to produce a more intense banding pattern earlier in the experiment (for example, compare lane 2A to lane 3A). These observations are consistent with the idea that these proteins result from the induction of plasmid genes. While the pDKE6 samples in lanes 4A-5B clearly show a band corresponding to the predicted size of 13,153 Da for the B-18 phasin, there are two potential bands that could represent the PHA synthase in lanes 2A-5B. There is a less intense band located slightly above the 66.2-kDa marker and a very intense band located slightly below the same marker. These two proteins are indicated as PHA synthase A and PHA synthase B, respectively. From the DNA sequence, there are two ATG codons in the *phaC*_*B18*_ gene that could potentially serve as transcriptional start sites. Both of these have an upstream sequence that could serve as a Shine-Dalgarno sequence or ribosome-binding site. The first codon contains a putative Shine-Dalgarno sequence (AGGAG) located twelve nucleotides upstream and produces a protein with the predicted size of 67,306 Da, while the second codon contains a putative Shine-Dalgarno sequence (AAGG) located eight nucleotides upstream and produces a protein with the predicted size of 62,675 Da. One possible explanation for these observations is that the induction conditions so overwhelms the cell with plasmids that the incorrect ATG codon (the second one) is used as the start of the gene. The presence of the *phaP*_*B18*_ gene provides the correct spacing so that the first ATG codon can be used as the translational start producing a functional PHA synthase enzyme.

To test which of these ATG codons could serve as the transcriptional start site for the *phaC*_*B18*_ gene and to determine if the phasin encoded within *phaP*_*B18*_ was necessary for PHA granule formation in enteric bacteria, a total of six mutations in pDKE6 were generated using PCR technology. As listed in Table 1, three mutations were created in the *phaC*_*B18*_ gene. Plasmid pDK108 contains a “double” nonsense mutation that replaces the first ATG codon and the second codon for two stop codons. Plasmid pDK201 contains a missense mutation that replaces the second methionine codon for an alanine codon. Plasmid pDK401 is a silent mutation that replaces the second potential Shine-Dalgarno sequence with degenerate codons to allow for the wild type amino acid sequence in the protein product. Table 1 also shows that three different knock-out mutations were created within the *phaP*_*B18*_ gene. Rather than creating a large deletion or insertion within *phaP*_*B18*_, these mutations allowed the spacing between *phaP*_*B18*_ and *phaC*_*B18*_ to remain essentially the same as in the parent plasmid, pDKE6. Plasmids pDK602 and pDK702 both contain early nonsense mutations while plasmid pDK802 contains a frameshift mutation located after the fourth codon in the gene to generate a nine-amino-acid peptide (M-Y-T-D-L-L-Q-N-F) with a predicted molecular weight of 1144 Da instead of the complete *phaP*_*B18*_ gene product. This nine-amino-acid peptide should not be visible in the SDS-PAGE analysis.

**Fig 4:**
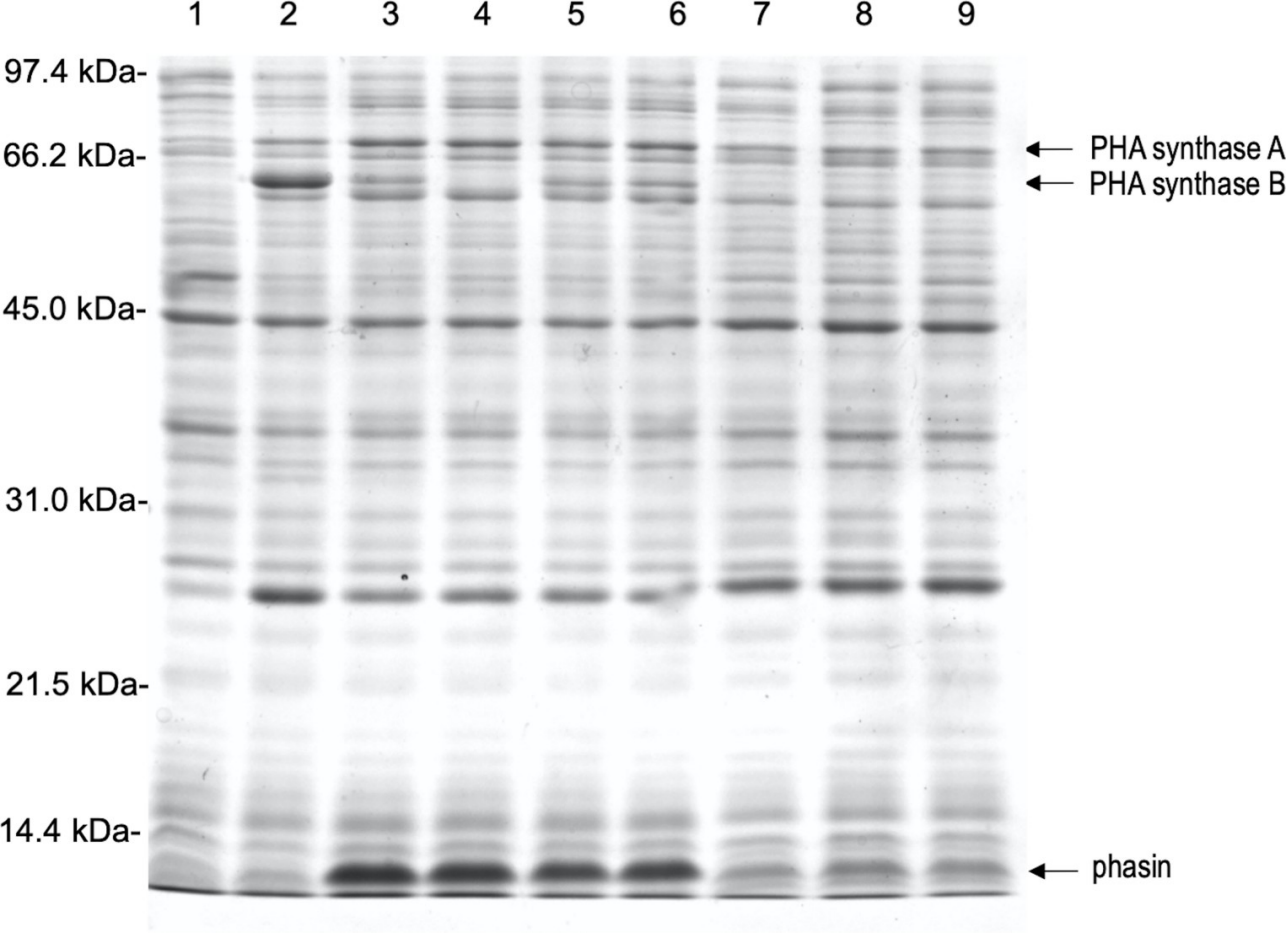
Expression of wildtype and mutant B-18 PHA genes using runaway replication in *E*. *coli* XL1-Blue. Samples 1: (pDKF2) 30°C, 2: (pDKF2) 41°C, 3: (pDKE6) 41°C, 4: (pDK108) 41°C, 5: (pDK201) 41°C, 6: (pDK401) 41°C, 7: (pDK603) 41°C, 8: (pDK702) 41°C and 9: (pDK802) 41°C. All samples were incubated 3 hours past the induction point. The sample volume was adjusted so that each lane contained the same amount of protein. The arrows indicate the probable location of PHA synthase (67.306 kDa or 62.675 kDa depending upon the location of the translational start) and the phasin (13.153 kDa predicted size).

Similar expression studies involving SDS-PAGE were performed with *E*. *coli* containing pDKF2, pDKE6, or one of the six mutant plasmids. For these experiments, only thermal induction was employed, and samples were collected at three hours post induction. Uninduced cells containing pDKF2 were also included in this analysis. In a similar fashion to the previous figure, arrows in Fig 4 show locations of induced proteins that correspond to the predicted sizes for both the two potential PHA synthases and the phasin. The intense band corresponding to “PHA synthase B” is missing from cells that lack the first ATG codon in *phaC*_*B18*_ (pDK108, lane 4), but is present in cells containing the parent plasmid (pDKE6, lane 3) as well as cells containing one of the two other *phaC*_*B18*_ mutant plasmids (pDK201, lane 5; pDK401, lane 6). The band corresponding to “PHA synthase A” is found in equal intensity in all of the lanes containing induced samples (lanes 2–9). Taken together, this suggests that the first ATG codon in *phaC*_*B18*_ is used as the start codon. We cannot explain why the apparent protein band corresponding to the PHA synthase is smaller in size than that predicted by the gene. The intense band corresponding to the B-18 phasin is seen in cells containing the parent plasmid (pDKE6, lane 3) and in cells containing one of the three *phaC*_*B18*_ mutations (lanes 4–6) but is missing in cells containing the pDKF2 control (lane 2) or any of the three *phaP*_*B18*_ mutant plasmids (lanes 7–9). Interestingly, an intense band corresponding to the *phaC*_*B18*_ gene product (“PHA synthase B”) is absent in these samples (lanes 7–9) as well.

Overall, the SDS-PAGE analysis confirmed that thermal induction leads to an overexpression of the B-**5**18 genes carried on the runaway replication vectors. It is interesting to note that the levels of PHA synthase were reduced in induced cells if the gene was located further from the transcriptional start (compare lanes 2 and 3 in Fig 4). This may simply reflect the location of the gene with respect to the promoter as it was shown that the gene order within an operon influences the level of transcription and translation [43] or this could have resulted from phasin activity. Perhaps the absence of a functional B-18 phasin in cells containing one of the three *phaP*_*B18*_ mutant plasmids (lanes 7–9 in Fig 4) further serves to reduce the levels of transcription of the *phaC*_*B18*_ gene.

### PHA accumulation

**Fig 5:**
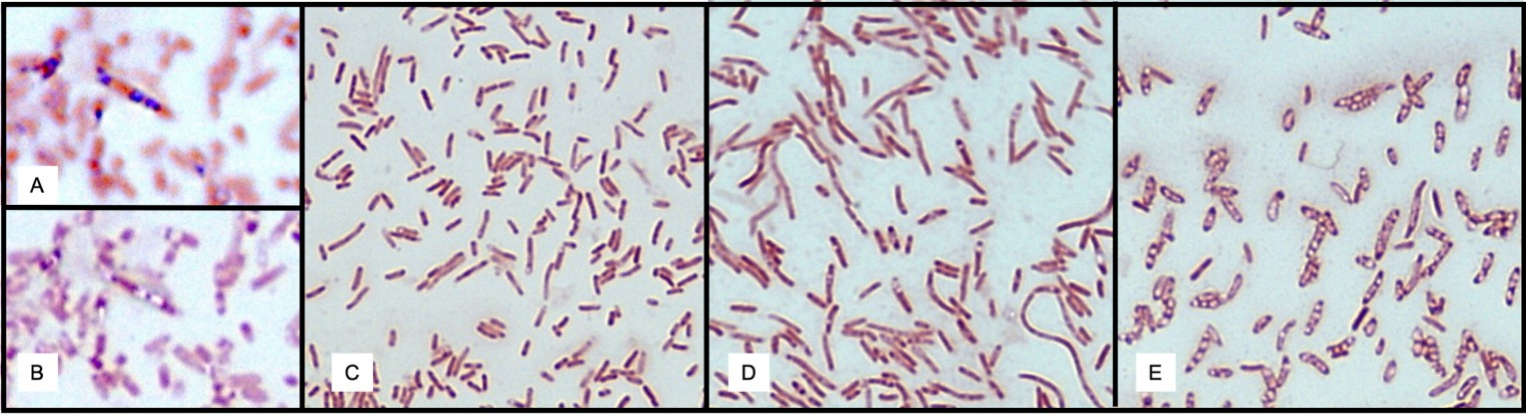
Microscopic observation of PHA accumulation. A: PHA granules stained with Sudan black; B: same slide with fine focus adjustment showing PHA as clear inclusions; panels C-E: representative slides showing the scale used to indicate PHA accumulation; C: no PHA inclusions visible (“-”); D: PHA inclusions seen in some, but not all, cells (“+/-”); and E: PHA inclusions seen in nearly every cell (“+”). In this figure, all panels show *E*. *coli* containing pUMS and one of the pDK-series plasmids.

In order to determine if the cloned B-18 genes allow for PHA accumulation, it was necessary to first supply the other genes needed for PHA accumulation in enteric bacteria. Both *phaA*_*Cn*_ and *phaB*_*Cn*_ were supplied *in trans* on a compatible plasmid. In the PHA accumulation studies, cells were first cultured in rich medium and harvested by centrifugation prior to resuspension in a minimal medium that was supplemented daily with glucose. Cells may be induced thermally and/or chemically at this time. This two-stage culturing method allows for a healthy cell population prior to subjecting the cells to the conditions that promote PHA accumulation but may not favor cell growth. Samples were taken daily and were used to prepare slides. Originally, the slides were stained for PHA with Sudan Black using standard techniques [41]. However, this technique did not provide consistent results (Fig 5, compare panel A to panel B). Instead, simple staining with safranin was sufficient to visualize the PHA granules, which were easily seen as clear inclusions within cells under oil immersion using a compound light microscope. The following subjective scale was used to judge the presence of PHA granules in the cells. Cells that did not contain granules were indicated by a “-“. A “+/-”was used to indicate that granules were seen in some cells, but not all. A “+” indicated that granules could be seen in nearly every cell. Representative examples illustrating this scale can be found in Fig 5 (panels C-E). Initial experiments were performed for up to four days and included samples that were subjected to no induction, chemical induction alone, thermal induction alone, and both chemical and thermal induction. For simplicity and clarity, Tables 3–5 only contain data from the first two days as the extended incubation time did not alter PHA accumulation in the cells.

**Table 3:** Microscopic observation of PHA granules in *E*. *coli* DH5α (pUMS, pDK-plasmid).

Condition	Day	F2	E6
30°C	1	-	+
	2	+/-	+
30°C + IPTG	1	-	+
	2	-	+
41°C	1	-	+/-
	2	-	+/-
41°C + IPTG	1	-	-
	2	-	+

(-): PHA granules were absent; (+/-): PHA granules were found in some, but not all cells; (+): PHA granules were found in nearly every cell. Plasmid pDKF2 contains *phaC*_*B18*_ and pDKE6 contains *phaP*_*B18*_*-phaC*_*B18*._

**Table 4:** Microscopic observation of PHA granules in *E*. *coli* DH5α (pUMS, pDK-plasmid).

pDK-series plasmid	Day 1–30°C	Day 2–30°C	Day 1–41°C	Day 2–41°C
108	-	-	-	-
201	-	+/-	-	-
401	+	+	+/-	-
602	-	-	-	-
702	-	-	-	-
802	-	-	-	-

(-): PHA granules were absent; (+/-): PHA granules were found in some, but not all cells; (+): PHA granules were found in nearly every cell.

**Table 5:** Microscopic observation of PHA granules in enteric bacteria (pUMS, pDK-plasmid).

Organism	pDK-series plasmid	Day 1–30°C	Day 2–30°C	Day 1–41°C	Day 2–41°C
*K*. *aerogenes*	F2	-	-	-	-
	E6	+	+	+/-	+/-
*S*. *flexneri*	F2	-	-	-	-
	E6	+	+	-	-

(-): PHA granules were absent; (+/-): PHA granules were found in some, but not all cells; (+): PHA granules were found in nearly every cell.

Table 3 contains the results from *E*. *coli* (pUMS) containing either pDKF2 or pDKE6. Cells can accumulate PHA more readily if an intact *phaP*_*B18*_ gene is present and can do so at the lower noninducing temperature. The addition of IPTG did not enhance PHA accumulation at the lower noninducing temperature and appeared to delay PHA accumulation at the higher inducing temperature.

Because the addition of IPTG did not significantly alter PHA accumulation in the cells (as shown in Table 3), later experiments focused on solely on the effect of thermal induction. Table 4 contains the results from *E*. *coli* (pUMS) plus one of the mutant pDK mutant plasmids. PHA was not detected in cells lacking the first ATG codon in *phaC*_*B18*_ (pDK108 in Table 4), confirming that this codon is the transcriptional start. Cells containing the silent mutation in *phaC*_*B18*_ (pDK401 in Table 4) showed similar PHA accumulation to cells containing the parent pDKE6 plasmid, while those containing a missense mutation involving the second ATG codon (pDK201 in Table 4) showed reduced PHA accumulation. The location of this second methionine is found within a conserved region found within many of the PhaC_B18_ homologs (results not shown) indicating that this amino acid residue may be crucial for enzyme function. To test the hypothesis that the B-18 phasin is crucial for PHA accumulation, three separate *phaP*_*B18*_ mutations in pDKE6 were generated. To ensure that the results reflected the absence of the B-18 phasin and not the relationship of the gene with respect to the promoter (as could be the case with pDKF2), these mutations essentially maintained the distance between the promoter and the transcriptional start of *phaC*_*B18*_. No PHA was detected in cells containing any of these *phaP*_*B18*_ mutant plasmids (pDK602, pDK702, or pDK802 in Table 4). While this appears to support the hypothesis that the B-18 phasin is necessary for enhanced PHA accumulation, we cannot rule out the possibility that these results reflect an absence of *phaC*_*B18*_ expression in cells containing one of the *phaP*_*B18*_ mutant plasmids as an intense band corresponding to PhaC_B18_ cannot be seen in the SDS-PAGE analysis (Fig 4, PHA synthase B in lanes 7–9).

To determine if the B-18 phasin enhances PHA accumulation in other enteric bacteria, similar microscopy experiments were performed in *S*. *flexneri* and *K*. *aerogenes*. Since neither of these organisms can accumulate PHA, it was necessary to supply the *phaA* and *phaB* genes *in trans* using pUMS. The results in Table 5 are similar to that seen with *E*. *coli* carrying the same plasmids (Table 3). Taken together, these observations indicate that the presence of the B-18 phasin allows the enteric hosts to more readily accumulate PHA at the lower (noninducing) temperature.

## Discussion

In this study, a runaway replication vector was chosen to control the expression of the PHA synthase (*phaC*_*B18*_*)* gene from a marine bacterium in the presence or absence of a phasin gene because plasmid genes may be easily overexpressed through either chemical or thermal induction [34]. The genes for the other enzymes needed for PHA synthesis in enteric bacteria, *phaA*_*Cn*_ and *phaB*_*Cn*_, were carried on a compatible plasmid and the levels of expression for these genes were presumed to be constant regardless of the incubation temperature. This differs from a similar study [35] where all three genes from *C*. *necator* were carried on runaway replication vectors. In particular, the study described in this article focused on the possible influence of a phasin gene on PHA accumulation in heterologous hosts.

Runaway replication has been employed as a means to overexpress genes carried on plasmids [for example: 30–36]. This technology has been successfully adapted for large scale production of proteins in fermentation reactors [30–32]. In addition, the runaway replication system can be modified to eliminate the need for antibiotics [36] and to allow for more stringent control [32, 35]. While these modifications serve to optimize gene expression in cost effective manner, it is fortuitous that the runaway replication vectors used in this study did not contain similar modifications. Although the overexpression of the PHA synthase gene was clearly induced by the higher incubation temperature (Figs 3 and 4), microscopic observation revealed that cells readily accumulate PHA at the noninducing (lower) temperatures, especially if the phasin gene was present (Tables 3–5). This suggests that low levels of transcription occurred at the noninducing temperature and these levels were sufficient to allow for PHA accumulation. While *pha* overexpression has led to increased PHA accumulation in *E*. *coli* [for example, 35], this observation is consistent with other studies that have shown *phaC* overexpression does not always correlate with increased PHA accumulation [for example 44]. Simultaneous expression of *phaP* and *phaC* can lead to enhanced PHA synthase activity, especially if both genes are derived from the same organism [45–47, reviewed in 27]. With these constructs, eliminating the need for either chemical or thermal induction could serve to further reduce production costs.

In some cases, weak PHA accumulation was seen for both *E*. *coli* and *K*. *aerogenes* carrying the *phaP*_*B18*_ phasin gene in cells incubated at 41°C (Tables 3 and 5). This is consistent with the evidence suggesting that phasins can act as chaperonins to ensure the proper folding of the PHA synthase and perhaps other proteins [45, 48–50]. Additionally, it has been suggested that phasins directly enhance the activity of PHA synthase by binding to the growing PHA molecule to prevent blockage of the enzyme release site [50]. The same observations would also occur if one or several enzymes involved in PHA synthesis do not function properly at the higher temperature used to induce runaway replication. Given that PHA accumulation occurred in thermally induced cells containing the *C*. *necator pha* genes carried on a runaway replication plasmid in an earlier study [35], the B-18 *pha* genes are the most likely candidates if this is the case. In either scenario, perhaps the B-18 phasin allowed for some functional PHA synthase to occur at the higher temperature.

There are several examples where phasins enhance PHA synthase activity and PHA accumulation in recombinant hosts [for example 46, 47, 51, 52; reviewed in 27]. There is also evidence suggesting that the heat shock protein, HspA, can substitute for the *C*. *necator* phasin (Php1) in recombinant cells containing *pha*_*Cn*_ genes [53] and explains why PHA was easily detected in the absence of a phasin in the original studies [for example 18]. The addition of the Php1 gene significantly increased PHA accumulation for cells carrying the *phaABC*_*Cn*_ genes [53]. There is some evidence to suggest that this PHA-synthase enhancing effect by phasins may be species-specific [52]. It would follow then that endogenous heat shock proteins cannot always substitute for a phasin and the enhancing effect may only occur in the presence of a specific phasin.

This study provides additional evidence that phasins enhance PHA accumulation and this enhancement was demonstrated in three different enteric hosts (Tables 3–5). In a recent study [29], 118 species were found to include a similar *pha* gene arrangement to that seen for B-18 (Fig 1). The majority of these are members of either the genus *Vibrio* or the genus *Photobacterium* and could represent a novel PHA gene cluster that is conserved among marine Gammaproteobacteria. BLAST searches revealed that many of these organisms contain homologous genes to *phaP*_*B18*_ and *phaC*_*B18*_ (partially shown in Fig 2) suggesting that B-18 should be included within this group. While the specificity of the enhancing effect of the B-18 phasin is not known, significant PHA accumulation in recombinant hosts containing PHA synthase genes from any of the organisms in this cluster is predicted to occur only in the presence the appropriate phasin gene.

## Supporting information

S1 Raw images (TIFF)
