# Supplementary figures and images for: Using runaway replication to express polyhydroxyalkanoic acid (pha) genes from a novel marine bacterium in enteric bacteria: The influence of temperature and phasins on PHA accumulation
Source: PLoS One. 2022 Dec 7;17(12):e0275597. doi: 10.1371/journal.pone.0275597 (PMC9728866; doi:10.1371/journal.pone.0275597)

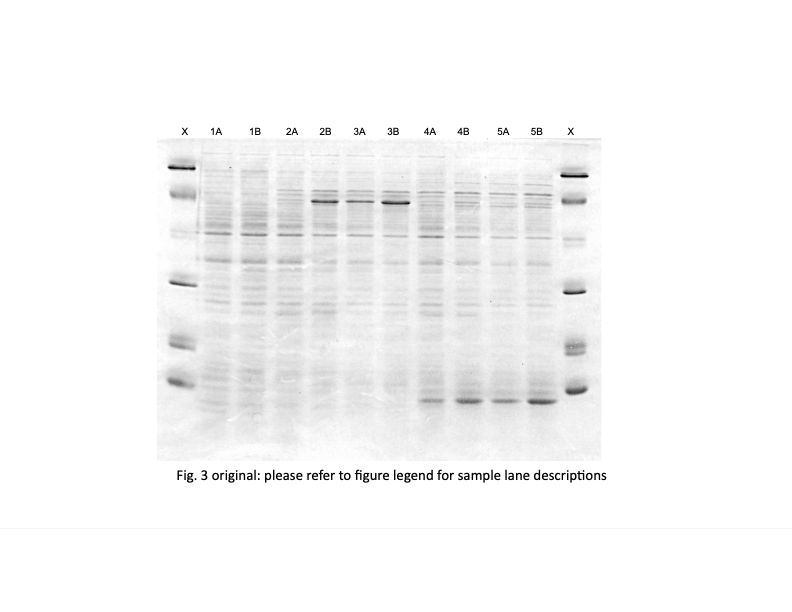

Supplement: S1 Raw images — (TIFF) [file pone.0275597.s001.tiff]
